# Avoiding artefacts in MicroCT imaging of collagen scaffolds: Effect of phosphotungstic acid (PTA)-staining and crosslink density

**DOI:** 10.1016/j.bioactmat.2021.06.012

**Published:** 2021-06-18

**Authors:** Kyung-Ah Kwon, Daniel V. Bax, Jennifer H. Shepherd, Ruth E. Cameron, Serena M. Best

**Affiliations:** a Cambridge Centre for Medical Materials, Department of Materials Science and Metallurgy, University of Cambridge, Cambridge, United Kingdom; b School of Engineering, University of Leicester, Leicester, United Kingdom

## Abstract

X-ray micro-computed tomography (μ-CT) can be used to provide both qualitative and quantitative information on the structure of three-dimensional (3D) bioactive scaffolds. When performed in a dry state, μ-CT accurately reflects the structure of collagen-based scaffolds, but imaging in a wet state offers challenges with radiolucency. Here we have used phosphotungstic acid (PTA) as a contrast agent to visualise fully hydrated collagen scaffolds in a physiologically relevant environment. A systematic investigation was performed to understand the effects of PTA on the results of μ-CT imaging by varying sample processing variables such as crosslinking density, hydration medium and staining duration.

Immersing samples in 0.3% PTA solution overnight completely stained the samples and the treatment provided a successful route for μ-CT analysis of crosslinked samples. However, significant structural artefacts were observed for samples which were either non-crosslinked or had low levels of crosslinking, which had a heterogeneous interior architecture with collapsed pores at the scaffold periphery. This work highlights the importance of optimising the choice of processing and staining conditions to ensure accurate visualisation for hydrated 3D collagen scaffolds in an aqueous medium.

## Highlights

• Important new insights on artefact prevention in scaffold imaging.
• First time μ-CT imaging of a **hydrated** collagen scaffold is reported.
• Optimisation of the use of phosphotungstic acid in μ-CT imaging.
• Correlation between crosslinking degree and artefact creation is established.
• A solution to processing and staining issues is highlighted.

## Graphical abstract

**Figure:**
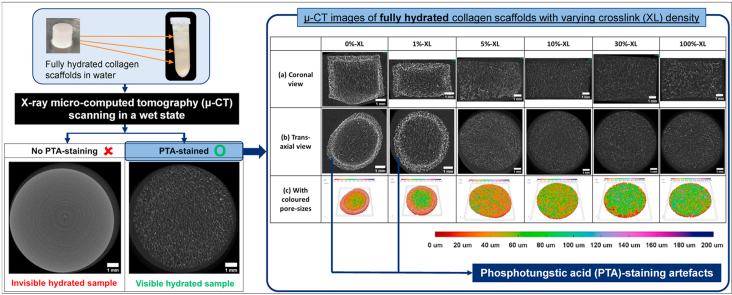

## 1 Introduction

Collagen is one of the most abundant proteins in the human body, forming a structural network to support cells *in vivo* [1]. This makes collagen the material of choice for three-dimensional (3D) scaffolds. 3D collagen scaffolds have been utilised in a wide range of potential applications including tissue regeneration [2,3], as a bioreactor substrate for the generation of blood platelets [4], as a 3D model for human tissues [5], and as a drug delivery vehicle [6]. The architecture of 3D collagen scaffolds is of great importance since structural characteristics such as mean pore size and construct porosity have been reported to significantly affect cellular activity [7,8], and mechanical [9] and fluid transport [10] properties. Hence, it is important to develop visualisation techniques that accurately represent the internal structure.

X-ray micro-computed tomography (μ-CT) is used extensively to characterise and analyse the internal structure of 3D scaffolds, but it is often performed in the dry state. However, collagen scaffolds become hydrated after implantation and therefore results obtained from dry samples do not necessarily give a true reflection of the structure *in vitro* or *in vivo.* Analysis of fully hydrated samples in aqueous conditions would provide more physiologically relevant data.

Although collagen scaffolds can provide good x-ray contrast in the dry state, a fully hydrated scaffold immersed in water cannot be visualised using μ-CT. This is because, as an organic material, collagen is mainly composed of low atomic number elements such as carbon, hydrogen and oxygen, which have comparable x-ray attenuation levels to water molecules. Treatments that add high atomic number elements are required to differentiate between the hydrated collagen and surrounding water.

Many researchers have utilised contrast agents that contain high atomic number elements such as osmium, tungsten and iodine, to improve x-ray attenuation of soft tissues in biological specimens. Examples include osmium tetroxide (OsO_4_) and phosphomolybdic acid (PMA) to visualise mouse embryos and molluscan soft tissue [11,12], iodinated contrast agent like Hypaque® 76 and Lugol's iodine solution (I_2_KI) to view mouse and rabbit brains as well as the alligator head of a post-hatchling [13,14], and phosphotungstic acid (PTA) to observe paddlefish heads and fly pupae [15]. The use of PTA has also been reported in the visualisation of hearts, lungs and liver in embryonic, postnatal and adult mice [16,17]. Collagen distribution in articular cartilage of horse and human was also investigated using PTA [18]. Amongst these contrast agents, PTA and iodine solution are the most commonly-used contrast agents due to their lower toxicity and greater availability. Different contrast agents result in somewhat different patterns of x-ray attenuation, PTA being shown to bind preferentially to connective tissues such as muscles, heart and lungs [11,15]. In addition, PTA was found to stain albumin, poly-ʟ-lysine, arginine, histidine and serotonin but failed to react with polyglutamic acid, glycogen, DNA and chondroitin sulfate A [19]. Quintarelli et al. [20] investigated the “staining mechanism” of PTA through a series of histochemical experiments and identified that PTA interacted primarily with organic cations by means of a salt type linkage, i.e. the heavy metal polyanion selectively binding to positively charged groups of the protein. The authors raised awareness on the importance of the presence of positively charged molecules in the substrates for PTA-binding to occur. Furthermore, the specific interaction between PTA and collagen was studied by Nemetschek et al. [21] who examined PTA-treated rat tail tendons under conventional and synchrotron X-ray radiation. The authors concluded that two specific reaction steps existed between PTA and collagen: the first step involving a firm intersubfibrillar binding of the polyanions PW_12_O_40_^3−^ to easily accessible basic groups that occurred within 10 min of processing. This was followed by the second step of less tight and intrasubfibrillar binding to relaxed fibres. The findings from these publications suggest suitability of PTA as a contrast agent to enhance the poor x-ray contrast of hydrated collagen scaffolds.

When collagen structures are created in the laboratory they do not contain the same degree of crosslinking present in native tissue. As a consequence, they exhibit limited structural stability and will degrade rapidly in a physiological environment. To overcome this issue, a chemical crosslinking step may be applied. For example, scaffolds can be chemically crosslinked, often using N-(3-Dimethylaminopropyl)-N′-ethylcarbodiimide hydrochloride) (EDC) and N-hydroxy-succinimide (NHS), which forms a zero-length amide bond between carboxylic acid and amine groups in collagen. Collagen can be crosslinked at varying degrees by varying molar ratio of EDC:NHS:COOH (on collagen). The ratio of 5:2:1 is standard in the literature and is often referred by the nominal term “100%-crosslinked”. The actual levels of crosslinking can be quantified by measuring the free primary amine content in collagen using 2,4,6-trinitrol-benzene-sulfonic acid (TNBS) or ninhydrin assay. The nominal degrees of crosslinking from 0% to 100% were reported to crosslink collagen at actual levels from 0% to 67% on average [2,22,23].

This EDC/NHS crosslinking has been shown to thermally stabilise collagen structures. An increase in shrinkage temperature from 56 °C to 86 °C was reported by Olde Damink et al. [24] when 100%-crosslinking dermal sheep collagen. Similarly, Lee et al. [25] crosslinked bovine pericardium with EDC:NHS at a 2:1 ratio, and noted an increase of denaturation temperature from 70 °C to 86 °C. Moreover, the EDC/NHS crosslinking was reported to enhance the mechanical stability of collagen scaffolds. For example, Davidenko et al. [22] found that the compressive Young's modulus of hydrated collagen scaffolds increased from 1.2 kPa to 6.2 kPa with increasing EDC/NHS crosslinking density (0.1%–100%). These thermal and mechanical improvements, however, come at the cost of cellular adhesion through ablation of cation-dependent integrin mediated cell attachment [22,26]. This means that, to maintain cell activity, crosslinking should be reduced to the minimum level required for a particular application. However, the effects of PTA staining on the structure of collagen scaffolds with varying degrees of EDC/NHS crosslinking are unknown.

In this paper, we report a systematic investigation of a range of sample processing variables to further understand the effects of PTA on μ-CT imaging of hydrated collagen scaffolds.

## 2 Materials

Insoluble microfibrillar bovine dermal Type I collagen powder was purchased from Devro Pty Ltd, Australia.

N-(3-Dimethylaminopropyl)-N′-ethylcarbodiimide hydrochloride) (EDC), N-hydroxy-succinimide (NHS), sterile-filtered Phosphate Buffered Saline (PBS) and phosphotungstic acid hydrate (PTA) were purchased from Sigma, UK.

## 3 Methods

### 3.1 Scaffold production

Collagen powder was swollen in 0.05 M acetic acid in a fridge at 4 °C for 72 h, to produce a 1% (w/v) collagen suspension. The swollen collagen was blended to make 1% (w/v) collagen slurry then vacuum degassed to remove any air bubbles that might have formed during the blending. The collagen slurry was pipetted into 6-well tissue culture plates (34.8 mm in diameter), with 8 mL added to each well. The slurry was then processed in a VirTis AdVantage freeze-drier (Biopharma Process Systems, UK). The slurry was frozen at −30 °C by cooling at 0.83 °C/min from 20 °C then the ice phase was sublimed under vacuum (80 mTorr) at 0 °C.

The fabricated collagen scaffolds were chemically crosslinked using EDC/NHS in ethanol/water (95% v/v) solvent at varying degrees (0%–100%), following the protocol by Olde Damink et al. [24] The 100%-crosslinking (100%-XL) was defined as crosslinking the collagen carboxylic acid groups (COOH) at molar ratios of EDC:NHS:COOH = 5:2:1. The crosslinking reaction was performed under ambient conditions for 2 h under gentle stirring, followed by washing in deionised water (DIW) for 75 min (five washes at 15 min each). The washed scaffolds were once again freeze-dried as described above.

Samples for immersion studies were prepared by first removing the top skin layer from the ~30 mm diameter freeze-dried collagen disc using a scalpel, then 8 mm-diameter and approximately 6 mm-thickness cylindrical samples were cut using an 8 mm-diameter biopsy punch.

### 3.2 Immersion studies

**Fig. 1:**
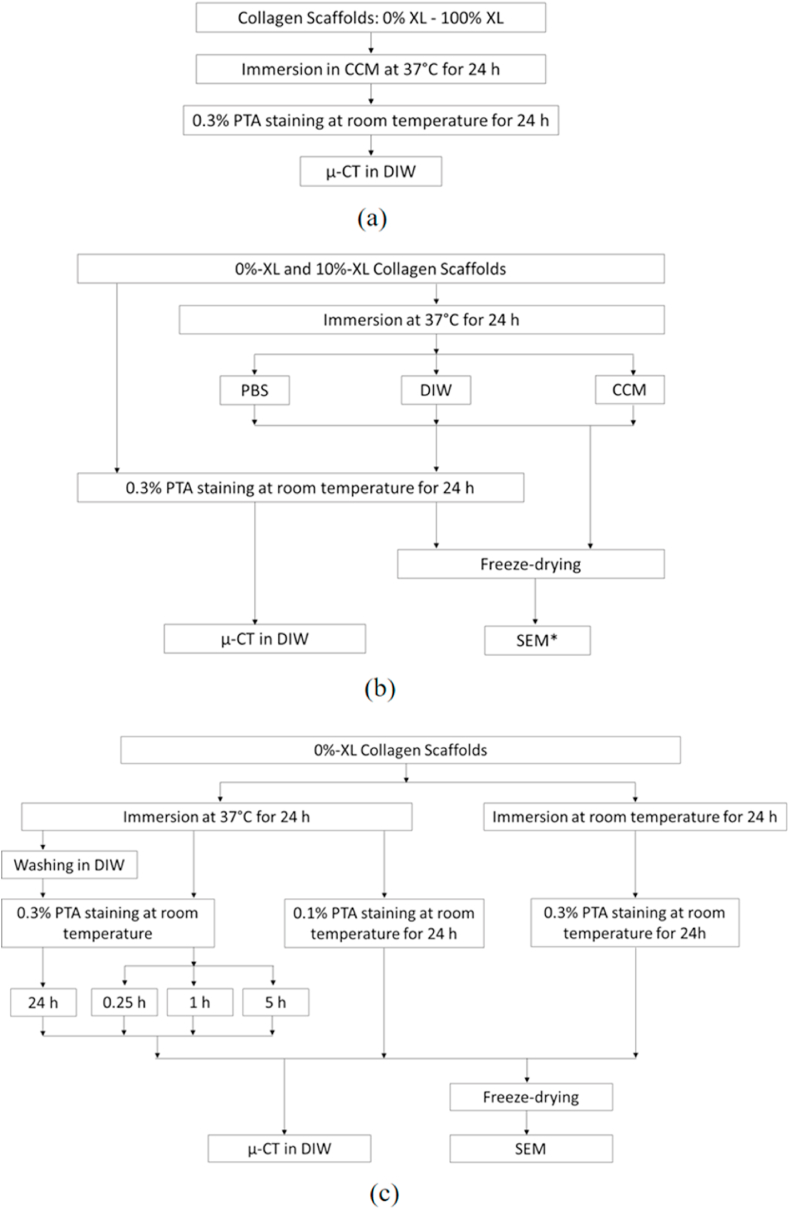
A series of immersion studies with their workflow (a) Immersion study I to investigate the effect of phosphotungstic acid (PTA)-staining on hydrated collagen scaffolds with varying crosslinking degrees (b) Immersion study II to examine the effect of different immersion medium prior to PTA-staining (c) Immersion study III to assess the effect of varying sample preparation methods for μ-CT in DIW. Notes: CCM = cell culture medium, PBS = phosphate buffered saline, DIW = deionised water, * in (b) is to note that SEM was only performed for 0%-XL scaffolds.

A series of immersion studies was performed as described below. The workflow of each immersion study is shown in Fig. 1.

#### 3.2.1 Immersion study I

This study was carried out to investigate the effect of PTA-staining on hydrated collagen scaffolds with varying degrees of crosslinking (XL). A fully supplemented cell culture medium (CCM) was chosen to simulate *in vitro* test conditions. CCM was prepared by adding 10% foetal bovine serum and 1% penicillin and streptomycin into Dulbecco's Modified Eagle's medium (DMEM, Sigma).

Collagen scaffold samples of varying degrees of XL (0%-XL, 1%-XL, 5%-XL, 10%-XL, 30%-XL and 100%-XL) were placed individually in each well of a 24-well tissue culture plate, containing 2 mL of CCM. The samples were kept in an incubator at 37 °C for 24 h. After incubation, the samples were prepared for μ-CT in DIW as described in Section 3.3.

#### 3.2.2 Immersion study II

The collagen scaffold samples of 0%-XL and 10%-XL were chosen to investigate the effect of immersion medium prior to PTA-staining.

Each collagen scaffold sample was immersed in 2 mL of CCM, PBS or DIW and kept in an incubator at 37 °C for 24 h. After incubation, the samples were prepared for μ-CT in DIW and SEM as described in Section 3.3 and Section 3.4, respectively.

Control samples were immersed in 0.3% PTA-solution and kept at room temperature (22.0 ± 0.6 °C) for 24 h. The samples were placed onto Rotamax 120 shaker (Heidolph Instruments, Germany) under a constant speed of 150 rpm to homogeneously distribute PTA-solution. After immersion, two samples were scanned in DIW using μ-CT, and two were freeze-dried and examined by SEM.

#### 3.2.3 Immersion study III

Additional experiments were conducted with 0%-XL collagen scaffolds samples to investigate the effect of varying sample preparation methods for μ-CT in DIW (Fig. 1c).

##### 3.2.3.1 Effect of washing to remove salt residue

After incubation in CCM at 37 °C for 24 h, the samples were washed in DIW with six changes of DIW every 5 min prior to 0.3% PTA-staining for 24 h under a shaking speed of 150 rpm.

##### 3.2.3.2 Effect of duration of PTA-staining

Samples were kept in the PTA-solution for 0.25 h, 1 h, or 5 h. In addition, the effect of PTA-solution concentration was assessed by staining the samples with 0.1% PTA-solution for 24 h under a shaking speed of 150 rpm.

##### 3.2.3.3 Effect of CCM temperature

The collagen samples were immersed in CCM at room temperature (22.0 ± 0.6 °C) for 24 h followed by 0.3% PTA-staining for 24 h under a shaking speed of 150 rpm.

### 3.3 μ-CT scanning

To perform μ-CT in DIW, a 0.3% (w/v) PTA-solution was prepared in DIW.

After 24 h-immersion in the respective media, each sample was removed and placed in 2 mL of the staining solution. PTA-staining was carried out at room temperature (22.0 ± 0.6 °C) for 24 h with shaking at a speed of 150 rpm. After staining, the sample was washed in 30 mL of DIW for 45 min (nine washes at 5 min each), then placed in an Eppendorf tube containing DIW. The sample was sandwiched between sponges to avoid any sample movement during scanning and the lid of the tube was kept tightly closed throughout scanning to prevent any water evaporation.

The Eppendorf tube, containing the stained sample in DIW, was placed on a sample holder in a μ-CT SkyScan 1172 (Bruker, Kontich, Belgium) and scanned using the following parameters: source voltage 60 kV, source current 167 μA, pixel size 5 μm, Al filter 0.5 mm, 180° rotation around the vertical axis of the sample with 2 average frames at every 0.2° angle step.

All μ-CT scanning was performed in duplicate.

### 3.4 μ-CT analysis

The raw scanned data were reconstructed using the NRecon software provided by Bruker. During reconstruction, the value of ring artefacts reduction was set to 20% and the value of beam-hardening correction was set to 30% for the PTA-stained samples. For the unstained control samples, the value of ring artefacts reduction was set to 10% and the value of beam-hardening correction was set to 0%.

The samples were viewed using the DataViewer software (Bruker) and a further 3D visualisation was obtained using the CTVox software (Bruker). For the latter, a volume of interest (VOI) of 1 mm-thick in the middle of each sample was selected by defining top and bottom surfaces and conducting a shrink-wrap procedure to automatically define the periphery of each sample in the CTAn software (Bruker). 3D analysis was performed to obtain pore sizes within the VOI. Subsequently, the VOI was visualised with colour-coded pore size distribution using CTVox (Bruker).

### 3.5 Scanning electron microscopy (SEM)

The surface morphology of scaffold samples before and after PTA-staining was examined using a SEM. The SEM samples were prepared by freeze-drying after immersing in respective medium. The freeze-drying protocol was as described in Section 3.1. The freeze-dried samples were sectioned in half, parallel to the top surface, using a scalpel, and then mounted on aluminium stubs with carbon tape and imaged at × 2000 magnification with a Phenom Pro 10 SEM, operating at 10 kV by backscattered electrons. No conductive coating was needed since a charge-reduction sample holder was used.

The overview of the sectioned scaffold samples was imaged at × 20 magnification using a FEI Nova NanoSEM, operating at 15 kV in secondary electron mode after sputter-coating the samples with a thin layer of palladium for 2 min at 20 mA.

## 4 Results

### 4.1 Immersion study I

**Fig. 2:**
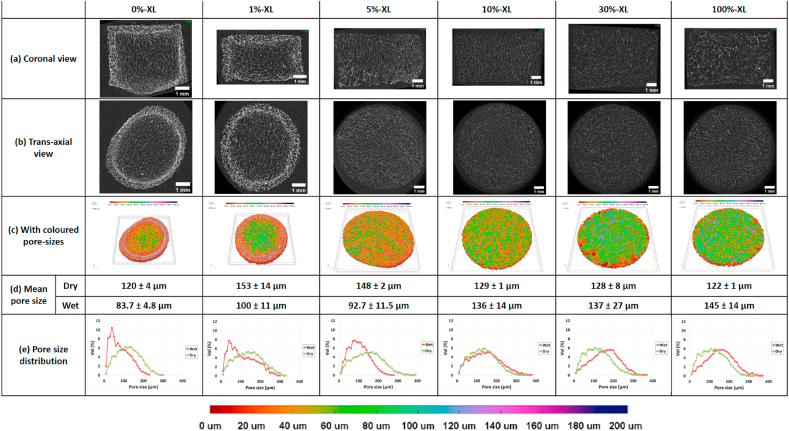
μ-CT images of collagen scaffolds with varying EDC/NHS crosslinking degrees (a) in coronal view (b) in *trans*-axial view (c) as volume rendered model of the scaffold structure superimposed with colour-coded pore-size distribution, (d) mean pore size and (e) pore size distribution of both dry and fully hydrated scaffolds. Scale bar in (a) and (b) represents 1 mm.

Fig. 2a and b shows the μ-CT images of PTA-stained, fully hydrated collagen scaffolds scanned in DIW viewed in different orientations. The internal structure of scaffolds was visualised and the pore sizes were evaluated. The pores in each scaffold were viewed as a volume rendered model of the scaffold structure superimposed with a colour-coded pore size distribution (Fig. 2c). The mean pore size and pore size distribution for both dry and fully hydrated scaffolds are also shown (Fig. 2d–e).

The hydrated scaffolds were scanned in duplicate and found to have highly porous interconnected porosity. The pore sizes appeared to be relatively homogenous throughout the bulk of scaffolds crosslinked with 5–100% EDC/NHS as shown by the unimodal pore size distribution (red lines in Fig. 2e). A densified region at the scaffold periphery was observed for 0%-XL and 1%-XL samples, consequently resulting in a change in shape of the pore size distribution. For these samples, the unimodal distribution (green lines in Fig. 2e) broadly changed towards a more bimodal distribution (red lines in Fig. 2e) after hydration with significant increase in the proportion of smaller pores in the total scaffold volume. As shown in the images with colour-coded pore sizes, this densified region comprised 10–20 μm diameter (red) pores.

### 4.2 Immersion study II

**Fig. 3:**
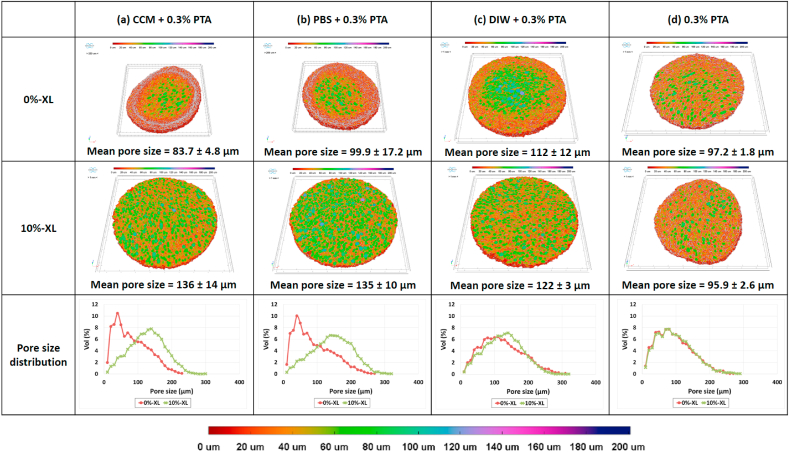
Mean pore size, pore size distribution and volume rendered models of 0%-XL and 10%-XL collagen scaffolds superimposed with colour-coded pore size distribution after immersing them in (a) cell culture medium (CCM) followed by 0.3% PTA staining (b) phosphate buffered solution (PBS) followed by 0.3% PTA staining (c) deionised water (DIW) followed by 0.3% PTA staining (d) 0.3% PTA staining alone. The immersion was for 24 h at 37 °C while the 0.3% PTA staining was for 24 h at ambient conditions.

Fig. 3 shows the volume rendered models of hydrated collagen scaffolds (0%-XL and 10%-XL) superimposed with colour-coded pore size distributions. The hydration was performed either in CCM, PBS or DIW for 24 h followed by 0.3% PTA-staining.

The 10%-XL samples showed a homogeneous structure irrespective of hydration media, while the 0%-XL samples had a heterogeneous structure, with smaller pores at the periphery of the scaffold. This collagen densification at the edge was more apparent for the samples immersed in CCM or PBS prior to staining than those immersed in DIW prior to staining, resulting in a more bimodal pore size distribution for the former samples.

Regardless of the crosslinking density, a homogeneous structure was seen for the control samples, which had been 0.3%-PTA stained alone.

**Fig. 4:**
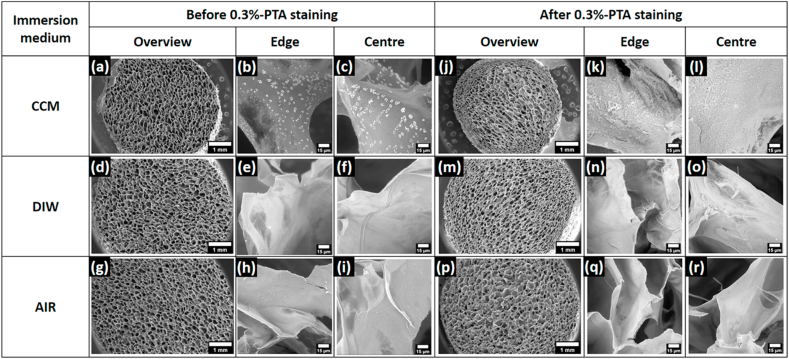
SEM images of 0%-XL collagen scaffolds before (a–i) and after 0.3%-PTA staining (j–r) for 24 h at ambient conditions. Prior to PTA-staining, the scaffolds were immersed in various media: cell culture medium (CCM: a-c, j-l) or deionised water (DIW: d-f, m-o) for 24 h at 37 °C, or kept in ambient conditions (g–i). Scale bar represents 1 mm for the overview images and 15 μm for the rest of images.

As 0%-XL scaffolds showed extensive densification, further structural assessment was conducted of these samples using SEM. To isolate the effect of PTA-staining, the scaffolds that had been immersed in various media were examined before and after PTA-staining (Fig. 4).

The overview SEM images of the stained samples (Fig. 4j and m) agreed well with the μ-CT results, showing a densified region with collapsed pores at the edge of the scaffolds. This occurred to varying degrees with more apparent densification for the stained sample following immersion in CCM than in DIW. The surface morphology for these samples appeared to be roughened and fibrous (Fig. 4k-l and Fig. 4n–o for immersion in CCM and DIW, respectively). Moreover, some precipitates were observed on the surface of the stained sample following immersion in CCM (Fig. 4k-l). As seen in the μ-CT images, a homogeneous structure was observed for the PTA-stained only sample (Fig. 4p). Unlike the other hydrated, stained samples, this showed a smooth surface morphology (Fig. 4q–r).

Prior to PTA-staining all samples exhibited smooth surfaces with uniformly distributed interconnected pores throughout their cross-sections regardless of immersion media. As such, the densified edge was not detected before PTA-staining (Fig. 4a and d). Some salt precipitates were seen on the smooth surface of the samples after immersion in CCM (Fig. 4b–c) but not DIW (Fig. 4e–f).

### 4.3 Immersion study III

**Fig. 5:**
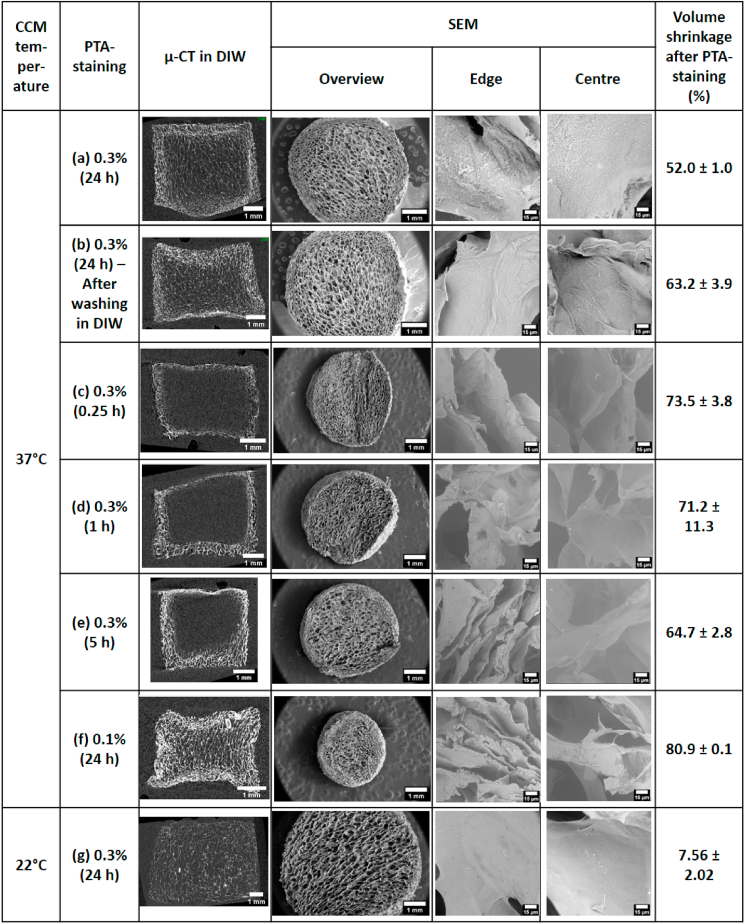
μ-CT and SEM images of 0%-XL collagen scaffolds after various sample preparation methods with respective volume shrinkage evaluated after PTA-staining (a) Immersion in CCM at 37 °C followed by 0.3%-PTA staining for 24 h (b) Immersion in CCM at 37 °C followed by washing in DIW for 30 min and 0.3%-PTA staining for 24 h (c) Immersion in CCM at 37 °C followed by 0.3%-PTA staining for 0.25 h (d) Immersion in CCM at 37 °C followed by 0.3%-PTA staining for 1 h (e) Immersion in CCM at 37 °C followed by 0.3%-PTA staining for 5 h (f) Immersion in CCM at 37 °C followed by 0.1%-PTA staining for 24 h (g) Immersion in CCM at 22 °C followed by 0.3%-PTA staining for 24 h. Scale bar represents 1 mm for the first two columns and 15 μm for the third and fourth columns.

Fig. 5 shows μ-CT and SEM images of 0%-XL scaffolds after various treatments. The percentage volume shrinkage of the scaffolds was evaluated and shown in Fig. 5.

#### 4.3.1 Varying the staining duration

The use of 0.3% PTA-staining for 15 min after scaffold immersion in CCM visualised only the outer boundary of the scaffold by μ-CT (Fig. 5c). The visible portion of the scaffold was found to increase with increasing staining duration (Fig. 5d–e). However, less than half of the sample was visible even after staining for 5 h (Fig. 5e), with a central core remaining unstained. The SEM images of these samples showed an increasing number of collapsed collagen layers at the edge with increasing staining time with no collapsed layer after 0.25 h-staining, 1–2 layers collapsed after 1 h-staining and approximately 5 layers of collagen pores collapsed after 5 h-staining. Regional variation in the surface morphology of these samples was also observed. While the centre of the samples had a smooth surface, the edge of the samples had textured and fibrous surface. About 52–74% volume shrinkage was observed with no clear trend between the staining duration and the level of volume shrinkage.

#### 4.3.2 Varying the sample preparation method for μ-CT in DIW

Different staining procedures were applied after immersion in CCM. The samples were either washed in DIW for 30 min prior to 0.3%-PTA staining or stained in 0.1%-PTA solution (Fig. 5b and f, respectively). These samples all exhibited similar structural alteration with a characteristic densified collagen region at the outer edges of the sample and collapsed pores observed in both μ-CT and SEM images. Moreover, the collagen struts exhibited a textured and fibrous surface in both cases throughout the scaffolds. It was noted that washing in DIW appeared to remove the precipitates seen in the stained sample after immersion in CCM (Fig. 5a vs. 5b). Increased volume shrinkage was observed when the DIW-washing step was introduced prior to PTA-staining and also when the concentration of PTA-solution was reduced from 0.3% to 0.1%.

However, unlike all the other samples investigated in Immersion study III, a homogeneous structure was seen for the PTA-stained sample following the immersion in CCM at 22 °C (Fig. 5g). There was no densified collagen region and the surface was smooth throughout the scaffold. Much lower volume shrinkage was observed than for the sample immersed at 37 °C.

## 5 Discussion

### 5.1 Effect of XL density

Staining fully hydrated collagen scaffolds in 0.3% PTA-solution allowed the visualisation of their internal structures in physiologically relevant environments, permitting subsequent structural analysis and predictions of cell infiltration into the scaffolds *in vitro* or *in vivo*. However, two different microstructures were observed by μ-CT (Fig. 2): a heterogeneous structure for non- and very weakly crosslinked scaffolds (0%-XL and 1%-XL) and a homogeneous structure for the others (≥5%-XL). The heterogeneous structure arose due to collapsed pores at the edge of the samples, thereby increasing the collagen density in this region and resulting in a broadly bimodal pore size distribution rather than a unimodal distribution.

During EDC/NHS crosslinking the carboxyl groups of aspartic (Asp) and glutamic (Glu) acid residues in collagen react with free amine groups of lysine (Lys), forming “zero-length” amide bonds between the collagen molecules [24]. This chemical crosslinking was reported to not only enhance the thermal stability of collagen by increasing the triple helix-to-random coil transition temperature (denaturation temperature) [24,25,27], but also to increase the structural stability by increasing elastic modulus with increasing XL density [22]. Our μ-CT results indicate that crosslinking collagen scaffolds (≥5%-XL) seems to provide sufficient thermal and mechanical strength to resist the structural alteration observed for the non- and very weakly crosslinked scaffolds (0%-XL and 1%-XL). Therefore, the crosslinking density is a crucial factor to consider during the sample preparation of hydrated scaffolds for μ-CT in aqueous environments.

### 5.2 Effect of immersion medium and PTA-staining

In searching for the possible reasons why such structural alteration was observed for non-crosslinked and very weakly crosslinked scaffolds, 0%-XL and 10%-XL scaffolds were selected to represent the heterogeneous structure and homogeneous structure, respectively. They were immersed in various media prior to PTA-staining. When the scaffolds were viewed as volume rendered models superimposed with colour-coded pore size distributions all of the samples showed a homogeneous structure except for the stained 0%-XL scaffolds following the immersion in CCM, PBS or DIW (Fig. 3a–c). It is worth noting that staining a 0%-XL scaffold in the PTA-solution alone resulted in a homogeneous structure with uniformly distributed pore sizes (Fig. 3d).

Further structural assessment was conducted for these 0%-XL samples using SEM (Fig. 4). Comparing the overview images of each scaffold before and after PTA-staining suggests that the densified collagen region around the edge of 0%- and 1%-XL scaffolds is likely to be an PTA-staining artefact because the densified collagen region was observed only after PTA-staining (Fig. 4j and m). No such structural alteration was detected for the freeze-dried scaffolds after immersing in aqueous media alone (Fig. 4a and d).

The mechanism of PTA-staining was reported by Silverman and Glick [19] who found that PTA primarily binds to positively charged protein groups such as poly-ʟ-lysine, arginine and histidine. In addition, Zhu et al. [28] reported that PTA in solutions decomposed in a stepwise manner with increasing pH from 1 to 8.3. The pH of 0.3%-PTA solution was measured to be 2.51 ± 0.09, implying that our staining solution are likely to contain [PW_12_O_40_]^3-^, [P_2_W_21_O_71_]^6-^ and [PW_11_O_39_]^7-^ ions based on Zhu et al.’s results [28]. EDC/NHS crosslinking consumes Lys, which means that crosslinked collagen scaffolds would have less positively charged groups available to react with the PTA anions. This loss of charged side chains, in addition to increased thermal and mechanical strength, could explain why the crosslinked collagen scaffolds (≥5%-XL) did not develop the staining artefact.

Staining dry collagen scaffolds did not result in significant structural changes even for 0%-XL samples: there was no densified region at the edge of the scaffold, both 0%-XL and 10%-XL scaffolds exhibiting almost the same unimodal pore size distribution (Fig. 3d). In addition, the surface remained smooth throughout the scaffold after the staining (Fig. 4q–r). However, staining a hydrated 0%-XL scaffold generated the staining artefact manifested by collapsed pores around the edge of the scaffold (Fig. 4j and m). Moreover, while the surface of the hydrated scaffold was smooth (Fig. 4b–c, 4e-f) it became textured and fibrous upon PTA-staining (Fig. 4k-l, 4n-o). Hydration and subsequent PTA-staining clearly led to the physical change. Upon further investigation, the choice of immersion medium showed a significant effect on the extent of the PTA-staining artefact. Greater densification of collagen was seen at the edge of the 0%-XL samples immersed in CCM or PBS than in DIW (Fig. 3a–b vs. Fig. 3c). This result suggests that the presence of salt in the medium weakens the collagen stability, thereby generating a more pronounced heterogeneous structure after PTA-staining. This agrees well with the findings by Privalov and Tiktopulo [29] who investigated the denaturation temperature of white rat skin tropocollagen. A decrease of the peak denaturation temperature from 40.5 °C to 37 °C was shown when these authors added 0.1 M NaCl into the collagen solution. This approximately equates to the 100 mM NaCl content of CCM and 150 mM NaCl content of PBS used here.

### 5.3 Methods for μ-CT scanning in DIW

In order to further investigate the effect of PTA-staining, various immersion and staining methods were applied to 0%-XL scaffolds (Fig. 1c), and the samples were characterised by μ-CT and SEM (Fig. 5).

Firstly, increasing the staining duration (Fig. 5c–e) demonstrated that staining occurs from the sample periphery towards the centre. This is as reported by Nemetschek et al. [21] who observed that rat tail tendon reacted with PTA from its periphery to the centre. These results indicate that PTA-collagen binding proceeds by the diffusion of PTA anions towards the scaffold centre.

Five hours was insufficient to completely stain a 5–6 mm thick scaffold with about 5 mm diameter (8 mm-diameter biopsied samples shrank after immersion in CCM). While no pore collapse was observed for the first 15 min of staining, with increasing staining duration, increasing numbers of pores collapsed at the scaffold edge, progressively increasing the collagen density. Furthermore, while the (yet unstained) centre of the scaffold had a smooth surface, the edge of the scaffold (PTA-stained) showed a textured and fibrous surface, suggesting an effect of PTA-staining on the surface morphology. Although no clear trend in volume shrinkage with staining duration was observed, about 50–75% volume shrinkage was seen during the staining.

Secondly, neither reducing the concentration of PTA solution nor introducing a washing step in DIW prior to staining removed the effect. The artefact associated with PTA-staining (collapsed pores at the edge of a scaffold) was seen for both cases (Fig. 5b and f). The washing step in DIW, however, removed the precipitation on the collagen surface (Fig. 5b vs. 5a). The precipitation on the stained collagen surface following immersion in CCM is attributed to the reaction between PTA anions and positively charged amino acids present in residual CCM. The PTA-staining was carried out by directly transferring the hydrated sample in CCM to PTA-solution. Hence, it is likely that the highly porous sample retained some residual CCM, which contains components including ʟ-arginine, ʟ-histidine and ʟ-lysine which can subsequently react with the PTA. Washing in DIW for 30 min prior to PTA-staining removes these residual CCM molecules, thereby preventing precipitation on the collagen surface (Fig. 5b).

The PTA-staining artefact was not detected for the 0%-XL scaffold which had been immersed in CCM at room temperature prior to the staining (Fig. 5g). Unlike the other PTA-stained scaffolds seen in Fig. 5, a homogeneous structure with no densified collagen region at the edge was seen for this sample. In addition, it had smooth surface throughout the structure with the least volume shrinkage (<10%). This result was somewhat surprising because the washed sample in DIW prior to the staining still exhibited heterogeneity with a roughened surface (Fig. 5b). Washing in DIW was performed at room temperature so there would have been no thermal gradient at the start of PTA-staining like the hydrated sample at room temperature. Nevertheless, the former resulted in a heterogeneous structure with roughened surface while the latter showed homogeneous structure with smooth surface. These results suggest that the hydration temperature prior to PTA-staining, in conjunction with crosslinking density, is a crucial factor for the formation of a densified region at the scaffold periphery. Regardless of the hydration medium, all of the 0%-XL scaffolds which had been hydrated at body temperature (37 °C) resulted in a heterogeneous structure with roughened surface after PTA-staining. This observation suggests that hydrating a 0%-XL scaffold at body temperature appears to cause an irreversible change in the steric arrangement, making the sample more susceptible to PTA-staining artefact (Fig. 5b).

Both pH and concentration of salt solution have been reported to affect the thermal stability of collagen. Usha and Ramasami [30] measured a decrease in the shrinkage temperature with rat tail tendon collagen when pH decreased from 8 to 5. The greater equilibrium swelling volume seen in more acidic environments was attributed to reduced thermal stability [[30], [31], [32]]. The pH of salt-containing solutions (CCM and PBS) with samples immersed was measured to be about 7 while that of DIW was about 4–5. This difference in pH in addition to the presence of salt might be why significantly greater volume shrinkage (about 50% vs. 10%) was observed for 0%-XL scaffolds (Fig. 3a–b vs. Fig. 3c). However, pH does not seem to affect the generation of PTA-staining artefacts because introducing an extra DIW-washing step prior to staining did not alleviate the artefacts (Fig. 5a–b) although the washing step would have reduced the pH of the solution that is retained in the collagen scaffolds (pH 7 to 5) in the same way as in the samples immersed in DIW prior to PTA-staining (Fig. 3c). Moreover, Komsa-Penkova et al. [33] reported a decrease in denaturation temperature in 5–450 mM NaCl solution and an increase in denaturation temperature in 450–1000 mM NaCl solution for acid-soluble calf skin Type I collagen, demonstrating the “salting-in/salting-out” effect. Although much greater volume shrinkage with more severe staining artefacts were observed for 0%-XL scaffolds in salt-containing solutions (Fig. 3a–b vs. Fig. 3c) no such artefact was observed for the samples immersed in CCM at room temperature (Fig. 5g). These observations further suggest that hydration temperature is much more critical than pH or the presence of salt in the formation of the densified region at the scaffold periphery.

When Privalov and Tiktopulo [29] took calorimetric measurements of rat-skin tropocollagen while recording optical activity, they observed a non-denaturation endothermic transition at around 33 °C as well as a denaturation endothermic transition at around 37 °C. Since hydrogen bond breaks endothermically, the 33 °C transition suggests the disruption of a fraction of H-bonds, but not to the extent of denaturation of the triple helix-to-random coil. Furthermore, Leikina et al. [34] demonstrated the potential for local microunfolding of collagen helices at below- or close to-body temperature. In line with these findings, it is possible that a non-denaturation thermal transition within 0%-XL scaffolds is occurring during hydration at 37 °C, exposing a greater number of positively charged amino acids on collagen to the surrounding solution. These ‘temperature exposed’ amino acids of collagen can subsequently react with PTA anions, resulting in more pronounced reaction than in the crosslinked samples. This may be linked to the surface roughening after PTA-staining.

One explanation for the location of this PTA artefact, at the scaffold periphery, is a potential PTA concentration gradient through the scaffold depth. At the initial stage of staining, the ratio of PTA anions to positively charged amino acids on collagen is high. However, PTA is lost (due to staining reactions) from the staining solution as it diffuses through the scaffold, resulting in a lower PTA anion to positively charged amino acid ratio in the scaffold centre. Hence, the effects arising from interaction with PTA would be greatest at the edge of the scaffold where PTA is most concentrated and would decrease with penetration depth through the scaffold due to a decreasing PTA concentration.

## 6 Conclusions

A simple method for staining hydrated collagen scaffolds has been reported which allows the 3D visualisation of their internal structure in aqueous medium using μ-CT. This method is useful for providing quantitative analysis under physiologically relevant conditions. However, this work has highlighted potential artefacts that can be created when staining non-crosslinked or very weakly crosslinked scaffolds (<5%-XL), associated with structural alterations at the periphery of the scaffolds. These changes in structure create a heterogeneous microstructure, which could lead to incorrect quantification of pore sizes. The optimisation of processing and staining conditions is therefore essential to ensure accurate tomographic visualisation and pore size analysis of 3D collagen scaffolds when hydrated in an aqueous medium.

## CRediT authorship contribution statement

**Kyung-Ah Kwon:** Conceptualization, Investigation, Writing – original draft, Visualization. **Daniel V. Bax:** Resources, Writing – review & editing. **Jennifer H. Shepherd:** Resources, Writing – review & editing. **Ruth E. Cameron:** Writing – review & editing, Supervision, Funding acquisition. **Serena M. Best:** Writing – review & editing, Supervision, Funding acquisition.

## Declaration of competing interest

None.

## References

[1] Shoulders M.D., Raines R.T. (2009). Collagen structure and stability. Annu. Rev. Biochem..

[2] Grover C.N., Cameron R.E., Best S.M. (2012). Investigating the morphological, mechanical and degradation properties of scaffolds comprising collagen, gelatin and elastin for use in soft tissue engineering. J. Mech. Behav. Biomed. Mater..

[3] Chan E.C., Kuo S.-M., Kong A.M., Morrison W.A., Dusting G.J., Mitchell G.M., Lim S.Y., Liu G.-S. (2016). Three dimensional collagen scaffold promotes intrinsic vascularisation for tissue engineering applications. PloS One.

[4] Shepherd J.H., Howard D., Waller A.K., Foster H.R., Mueller A., Moreau T., Evans A.L., Arumugam M., Chalon G.B., Vriend E., Davidenko N., Ghevaert C., Best S.M., Cameron R.E. (2018). Structurally graduated collagen scaffolds applied to the ex vivo generation of platelets from human pluripotent stem cell-derived megakaryocytes: enhancing production and purity. Biomaterials.

[5] Abbas Y., Brunel L.G., Hollinshead M.S., Fernando R.C., Gardner L., Duncan I., Moffett A., Best S., Turco M.Y., Burton G.J., Cameron R.E. (2020). Generation of a three-dimensional collagen scaffold-based model of the human endometrium. Interface Focus.

[6] Friess W., Lee G. (1996). Basic thermoanalytical studies of insoluble collagen matrices. Biomaterials.

[7] Riedl A., Schlederer M., Pudelko K., Stadler M., Walter S., Unterleuthner D., Unger C., Kramer N., Hengstschläger M., Kenner L., Pfeiffer D., Krupitza G., Dolznig H. (2017). Comparison of cancer cells in 2D vs 3D culture reveals differences in AKT-mTOR-S6K signaling and drug responses. J. Cell Sci..

[8] Murphy C.M., O'Brien F.J. (2010). Understanding the effect of mean pore size on cell activity in collagen-glycosaminoglycan scaffolds. Cell Adhes. Migrat..

[9] Al-Munajjed A.A., Hien M., Kujat R., Gleeson J.P., Hammer J. (2008). Influence of pore size on tensile strength, permeability and porisity of hyaluronan-collagen scaffolds. J. Mater. Sci. Mater. Med..

[10] Ahn G., Park J.H., Kang T., Lee J.W., Kang H.-W., Cho D.-W. (2010). Effect of pore architecture on oxygen diffusion in 3D scaffolds for tissue engineering. J. Biomed. Eng..

[11] Descamps E., Sochacka A., De Kegel B., Van Loo D., Van Hoorebeke L., Adriaens D. (2014). Soft tissue discrimination with contrast agents using micro-CT scanning. Belg. J. Zool..

[12] Golding R.E., Jones A.S. (2007). Micro-CT as a novel technique for 3D reconstruction of molluscan anatomy. Molluscan Res..

[13] de Crespigny A., Bou-Reslan H., Nishimura M.C., Phillips H., Carano R.A.D., D'Arceuil H.E. (2008). 3D micro-CT imaging of the postmortem brain. J. Neurosci. Methods.

[14] Gignac P.M., Kley N.J. (2014). Iodine-enhanced micro-CT imaging: methodological refinements for the study of the soft-tissue anatomy of post-embryonic vertebrates. J. Exp. Zool..

[15] Metscher B.D. (2009). MicroCT for comparative morphology: simple staining methods allow high-contrast 3D imaging of diverse non-mineralized animal tissues. BMC Physiol..

[16] Dullin C., Ufartes R., Larsson E., Martin S., Lazzarini M., Tromba G., Missbach-Guentner J., Pinkert-Leetsch D., Katschinski D.M., Alves F. (2017). μCT of *ex-vivo* stained mouse hearts and embryos enables a precise match between 3D virtual histology, classical histology and immunochemistry. PloS One.

[17] Lesciotto K.M., Motch Perrine S.M., Kawasaki M., Stecko T., Ryan T.M., Kawasaki K., Richtsmeier J.T. (2020). Phosphotungstic acid-enhanced microCT: optimized protocols for embryonic and early postnatal mice. Dev. Dynam..

[18] Nieminen H.J., Ylitalo T., Karhula S., Suuronen J.-P., Kauppinen S., Serimaa R., Haeggström E., Pritzker K.P.H., Valkealahti M., Lehenkari P., Finnilä M., Saarakkala S. (2015). Determining collagen distribution in articular cartilage using contrast-enhanced micro-computed tomography. Osteoarthritis Cartilage.

[19] Silverman L., Glick D. (1969). The reactivity and staining of tissue proteins with phosphotungstic acid. J. Cell Biol..

[20] Quintarelli G., Zito R., Cifonelli J.A. (1971). On phosphotungstic acid staining. I. J. Histochem. Cytochem..

[21] Nemetschek T., Riedl H., Jonak R. (1979). Topochemistry of the binding of phosphotungstic acid to collagen. J. Mol. Biol..

[22] Davidenko N., Schuster C.F., Bax D.V., Raynal N., Farndale R.W., Best S.M., Cameron R.E. (2015). Control of crosslinking for tailoring collagen-based scaffolds stability and mechanics. Acta Biomater..

[23] Nair M., Johan R.K., Hamaia S.W., Best S.M., Cameron R.E. (2020). Tunable bioactivity and mechanics of collagen-based tissue engineering constructs: a comparison of EDC-NHS, genipin and TG2 crosslinkers. Biomaterials.

[24] Olde Damink L.H.H., Dijkstra P.J., van Luyn M.J.A., van Wachem P.B., Nieuwenhuis P., Feijen J. (1996). Cross-linking of dermal sheep collagen using a water-soluble carbodiimide. Biomaterials.

[25] Lee J.M., Edwards H.H.L., Pereira C.A., Samii S.I. (1996). Crosslinking of tissue-derived biomaterials in 1-ethyl-3-(3-dimethylaminopropyl)-carbodiimide (EDC). J. Mater. Sci. Mater. Med..

[26] Bax D.V., Davidenko N., Gullberg D., Hamaia S.W., Farndale R.W., Best S.M., Cameron R.E. (2017). Fundamental insight into the effect of carbodiimide crosslinking on cellular recognition of collagen-based scaffolds. Acta Biomater..

[27] Yang C. (2012). Enhanced physicochemical properties of collagen by using EDC/NHS-crosslinking. Bull. Mater. Sci..

[28] Zhu Z., Tain R., Rhodes C. (2003). A study of the decomposition behaviour of 12-tungstophosphate heteropolyacid in solution. Can. J. Chem..

[29] Privalov P.L., Tiktopulo E.I. (1970). Thermal conformational transformation of tropocollagen. I. Calorimetric study. Biopolymers.

[30] Usha R., Ramasami T. (2000). Effect of pH on dimensional stability of rat tail tendon collagen fiber. J. Appl. Polym. Sci..

[31] Elden H.R. (1958). Rate of swelling of collagen. Science.

[32] Miles C.A., Burjanadze T.V., Bailey A.J. (1995). The kinetics of the thermal denaturation of collagen in unrestrained rat tail tendon determined by differential scanning calorimetry. J. Mol. Biol..

[33] Komsa-Penkova R., Koynova R., Kostov G., Tenchov B.G. (1996). Thermal stability of calf skin collagen type I in salt solutions. Biochim. Biophys. Acta.

[34] Leikina E., Mertts M.V., Kuznetsova N., Leikin S. (2002). Type I collagen is thermally unstable at body temperature. Proc. Natl. Acad. Sci. Unit. States Am..

